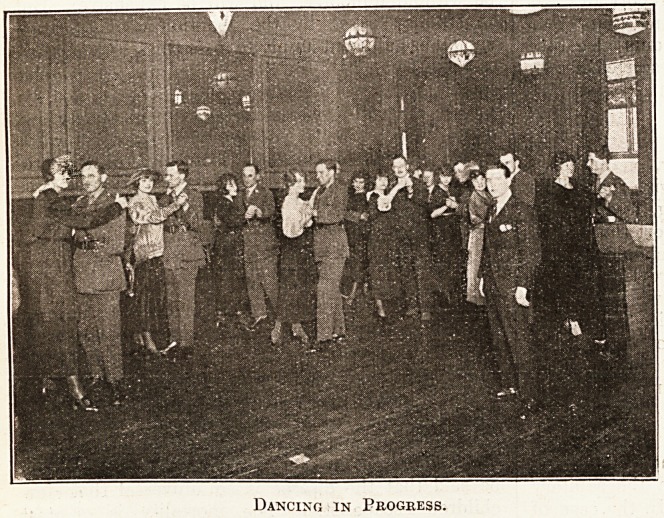# Dancing in Relation to the Use of Artificial Legs

**Published:** 1920-01-03

**Authors:** 


### DANCING IN RELATION TO THE USE OF ARTIFICIAL LEGS.

### A NOVEL AND INTERESTING DEVELOPMENT.

THE consequences of the war have resulted in the creation of many improvements in artificial legs, but all inventive genius in this direction is abortive in the absence of instruction in their use, as may be seen by comparing the utility of artificial legs prior to the war with the developments of the present day. There is no question that much attention has been bestowed upon the art of walking correctly with the aid of an artificial limb. The idea of dancing as well as walking is a novelty, and upon first consideration would appear to be merely a means of providing pleasure as well as the power of locomotion. This is not the case, however, for walking gracefully and dancing gracefully both depend upon bodily balance, and to adjust the bodily balance to altered circumstances must necessarily be the basis of instruction.

### THE ORIGINATOR.

The happy thought of teaching those who had lost a leg to dance again occurred to Mons. Jean Castener, the well-known dancer of the Adelphi Theatre. With the characteristic spirit of the artist he offered to teach the art of dancing with artificial limbs to those officers who have lost a leg and are under treatment in the Physico-Therapeutic Department of St. Thomas's Hospital, which is in the charge of Sister Randall. His offer was accepted by Sir Arthur Stanley with enthusiasm, and the progress which the famous dancer has made with his pupils was demonstrated at the Portman Rooms, Dorset Street, W., where amongst those who were present to witness this exceedingly interesting exhibition were Sir Arthur Stanley, Sir Arthur Lawley, and Major-General Sir A. Betheune. The secret of giving this instruction successfully, Mons. Castener explains, is to impress upon the pupil that the pivot of balance must be at the shoulders, and as an aid to the memory of this law the partner is instructed to place slight pressure with her hand upon the shoulder.

A DEMONSTRATION IN BALANCE.

DANCING IN PROGRESS.

The pupils commence by learning to walk forwards, backwards, and from side to side to the rhythm of music. By degrees they are able to accomplish the simple steps of the One-step and Fox Trot. This is the limit of progress so far. Waltzing and other more difficult dances will follow as progress is made. One of the most pleasing features of the exhibition is to witness the genuine enjoyment of the men who are under instruction. There can be no doubt that much physical benefit will be derived from this form of exercise; but perhaps more important still, as Mons. Castener especially emphasises, is the value of acquiring correct bodily balance as a means of preventing accidents in the streets, which so frequently overtake those who are handicapped by the loss of a leg. In any case the originators are to be congratulated on the success already achieved.

## Figures and Tables

**Figure f1:**
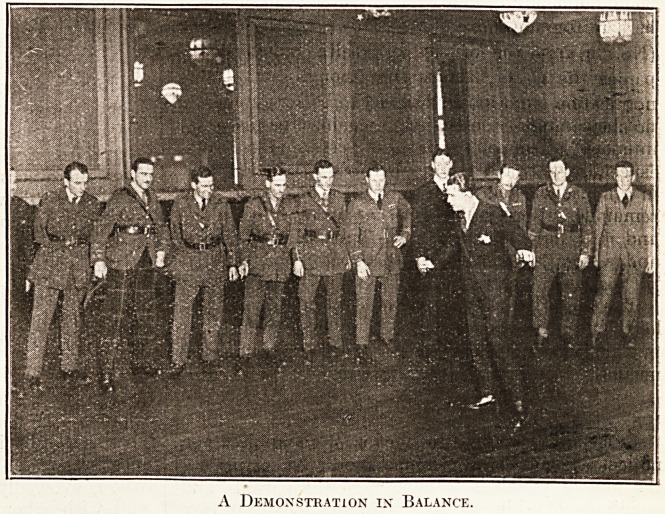


**Figure f2:**